# Seed Dormancy Release and Germination Requirements of *Cinnamomum migao*, an Endangered and Rare Woody Plant in Southwest China

**DOI:** 10.3389/fpls.2022.770940

**Published:** 2022-01-27

**Authors:** Jing-zhong Chen, Xiao-long Huang, Xue-feng Xiao, Ji-ming Liu, Xiao-feng Liao, Qing-wen Sun, Liang Peng, Lan Zhang

**Affiliations:** ^1^College of Forestry, Guizhou University, Guiyang, China; ^2^Guizhou Academy of Forestry, Guiyang, China; ^3^Guizhou Province Institute of Mountain Resources, Guiyang, China; ^4^College of Pharmacy, Guizhou University of Traditional Chinese Medicine, Guiyang, China

**Keywords:** regeneration, *Cinnamomum migao*, dormancy and germination trait, endogenous inhibitor, seed stratification

## Abstract

Seed dormancy is a complex adaptive trait of plants that are influenced by several physiological and environmental factors. The endangered plant *Cinnamomum migao* is also known to exhibit seed dormancy and low germination, which may influence its regeneration; however, these characteristics remain unexplored. To our knowledge, this study is the first to examine the type of dormancy and improve the germination percentage of *C. migao* seeds. We evaluated the structure and characteristics of the embryo and endocarp of *C. migao* seeds as well as the effects of endogenous inhibitors. Furthermore, we assessed the effects of light, stratification, alternating temperature, and gibberellic acid 3 (GA_3_) on the dormancy release of these seeds. The embryo was well developed the endocarp was water-permeable, and no obvious mechanical hindrance to germination was observed. However, the endocarp and embryo contained phenols and other germination inhibitors. The seed extracts of *C. migao* delayed the germination of cabbage and ryegrass seeds, which indicates the presence of endogenous inhibitors. These findings suggest that *C. migao* seeds exhibit physiological dormancy. Light and an alternating temperature (15/20°C) did not influence germination. However, GA_3_ pretreatment, alternating temperatures, and warm stratification relieved dormancy. GA_3_ pretreatment combined with the 15°C stratification treatment was most effective in rapidly releasing the *C. migao* seed dormancy. Our findings may facilitate the storage and conservation of this endangered plant, which is currently underrepresented in *ex situ* collections.

## Introduction

Seed dormancy describes the phenomenon in which seeds with vigor and integrity fail to germinate within a certain period even when the environmental conditions (e.g., water, light, temperature, and oxygen) are suitable (Finch-Savage and Leubner-Metzger, 2006; Yan and Chen, 2020). Seed dormancy is a physiological and ecological adaptation that enables plants to survive in complex environments. Dormancy also aids wild plant seeds in maintaining their vitality under harsh environmental conditions, thus contributing to plant survival, species continuation, and evolution (Stevens et al., 2020). Fresh seeds that do not germinate for 4–6 weeks under suitable conditions are considered dormant (Ensslin et al., 2018). Previous studies have estimated that 50–90% of wild plant seeds exhibit dormancy (Tang et al., 2004). According to the widely recognized classification system of Baskin and Baskin (2004), seed dormancy can be categorized into five classes: physiological dormancy (PD), morphological dormancy (MD), physical dormancy (PY), morphophysiological dormancy (MPD), and combinational dormancy (PY + PD). These classes are further categorized into subclasses, levels, and types (Baskin and Baskin, 2004).

Dormancy can prevent the seeds from germinating in unsuitable seasons, thus reducing species competition and ensuring population reproduction under adverse conditions (Rubio de Casas et al., 2012; Nonogaki, 2019). However, this trait poses a challenge when large numbers of seedlings are required for horticultural purposes, particularly for the conservation and rapid recovery of endangered plant populations (Cho et al., 2018; Gao et al., 2021). For the restoration of endangered populations in the wild, it may take several seasons for the seeds to become nondormant, which may result in low final germination percentages. Moreover, seeds are often lost during the dormancy period because of animal foraging, microbial attack, and soil erosion; this hinders the recovery of plant populations (Kildisheva et al., 2020; Ma et al., 2020; Zida et al., 2020).

*Cinnamomum migao* H. W. Li—an evergreen arbor belonging to Lauraceae—is a rare endemic species in China. Its distribution is limited to the area surrounding the dry hot valley formed by a small watershed in the Guizhou, Yunnan, and Guangxi Provinces in Southwest China (Li et al., 2013). *C. migao* has been classified as near-threatened in the Red List of Biodiversity in China: Volume of Higher Plants (Ministry of Ecology and Environment, P.R.C., 2011). It produces high-quality wood that is used for making high-grade furniture and wood carvings (Shi et al., 2003). The fruit is rich in sugars, amino acids, and volatile oils and is widely used as a spice and traditional medicine in Southwest China, particularly as a substitute for Lauraceae spices such as *Litsea lancilimba* and *Litsea cubeba* (Qiu and Du, 2010; Zhou et al., 2010; Wang et al., 2016). However, the wild population of *C. miga*o has been reported to be endangered and its natural regeneration faces various obstacles. The population of this arbor comprises adult trees aged >50 years and seedlings are extremely scarce. Previous evidence has confirmed seed dormancy in *C. migao* (Zhou et al., 2010; Huang et al., 2019); seed germination is very slow even in suitable environment, and the natural germination percentage is <36% (Gao et al., 2015). Our group further confirmed that seedlings germinated from *C. migao* seeds are scare in the wild (Li, 2017; Huang et al., 2019). The low germination percentage may result from seed dormancy, which prevents the supplementation *C. migao* seedlings. This is a primary barrier of natural regeneration of *C. migao*, ultimately making it an endangered species (Shi et al., 2003; Huang et al., 2021).

However, to our knowledge, the factors responsible for the low seed dormancy of *C. migao* and effective methods for dormancy release are unknown. This study, therefore, aimed to uncover the primary factors responsible for seed dormancy, such as anatomy, morphology, and physiology. The secondary objective was to explore the most effective method for *C. migao* seed dormancy release using alternating temperature, light/dark phase, gibberellic acid 3 (GA_3_), and clod/warm stratification. Our ultimate goal was to determine the type of seed dormancy and find a highly effective method to release seed dormancy of this arbor. Our findings may be a point of reference for future interventions aiming at raising seedlings *ex situ* and reintroducing them into the wild.

## Materials and Methods

### Fresh Seed Collection

*Cinnamomum migao* fruits were collected from Luodian County, Guizhou Province, China (25°26′40″N, 106°31′51″; 667 m altitude) in October–November 2017. The ripened fruits were transported to the laboratory immediately after collection. The fruits were then peeled, washed under running water, and placed in a ventilated dark room. The seeds enclosed by the endocarp were stored in the dark at 20 ± 2°C and approximately 60% relative humidity for 1 week before use.

### Morphology of Seeds and Embryos

The transverse axis diameter, longitudinal axis diameter, and thickness of *C. migao* seeds were measured using a vernier caliper (0.01 mm). The caliper was slowly clamped along the seed ridge to separate the embryo from the endocarp, and embryo development was observed and photographed under a fluorescence microscope (Leica M205 FA). Three replicates of 30 seeds were measured.

### Determination of Seed and Endocarp Characteristics

#### Seed Weight, Moisture Content, and Viability

In the beginning of December 2017, *C. migao* seeds were crushed and sieved through a No. 20 mesh screen, weighed accurately to 50 g, and oven-dried at 103°C for 17 h and then weighed again. Water content was determined based on the difference between fresh and dry weights. To test seed viability, embryos were placed in a 0.50% 2,3,5-triphenyltetrazolium chloride solution and then incubated in the dark at 25°C for 12 h. Embryos that had been kept in boiling water for 20 min for inactivation served as the control. Seed viability was determined based on the coloring position and depth of the embryos (Souza et al., 2010). Three replicates of 30 seeds each were used (Supplementary Figure S1: viability test results).

#### Imbibition Rate and Fruit Endocarp Characteristics

The seeds were categorized into three groups: first (control), seeds with the endocarp; second, seeds with partial endocarp (one-fifth of the endocarp was cut off); and third, seeds without the endocarp (Supplementary Figure S2). Seeds belonging to these three groups (50 seeds each) were placed in separate beakers with 200 ml of distilled water. They were then placed in a 25°C incubator and weighed using an electronic balance (0.0001 g) every 2 h until the weights stabilized. Before weighing, the water on the surface of the seeds was soaked using absorbent paper. Water absorption was calculated as the percentage of initial weight increase. For ultrastructural observation, the endocarps were fixed with 2.5% glutaraldehyde solution, washed with 0.1 M phosphate buffer, fixed with 1% osmic acid in 0.1 M phosphate buffer (pH 7.4) at room temperature (20°C) for 1–2 h, rinsed with 0.1 M phosphate buffer (pH 7.4), and then dehydrated using an ethanol gradient. The samples were dried in a critical point dryer (quorum K850) and sprayed with 30% gold using an ion-sputtering instrument (IXRF MSP-2S) for approximately 30 s. The endocarp ultramicrostructure was observed under a scanning electron microscope (Hitachi su8100).

#### Effects of H_2_O_2_, NaOH, and H_2_SO_4_ Treatments on Seed Germination

Toward the end of November, whole seeds were soaked in 15, 20, and 40% H_2_O_2_; 10, 20, and 40% NaOH; and 30, 50, and 98% H_2_SO_4_ for 10 min. Whole germination units (seed enclosed by endocarp, hereafter indicated as “seeds”) were soaked in distilled water. Subsequently, the seeds were washed under running water for 2 h, disinfected with 1% NaClO solution for 20 min at room temperature, and washed with sterile distilled water for the germination test (Chien and Lin, 1994). All seeds were disinfected after treatments to avoid seed contamination from the previous operation steps. Seeds were incubated at 25°C in dark and light (22.4 μmol m^−2^ s^−1^) at 40 ± 5% humidity; the treatment temperature was 25°C. Three replicates of 50 seeds each were used for the treatments.

### Inhibitory Effects of *C. migao* Seed Extract on Germination

#### Extraction Method

The endocarps and embryos were separated from the *C. migao* seeds in the end of November; these parts were then separated and frozen using liquid nitrogen. Next, 50 g of the endocarps and embryos were weighed and extracted using the continuous reflux method. On the basis of the polarity gradient of solvents—petroleum ether (30–60°C, polarity: 0.01) <acetic acid (polarity: 4.3) <acetone (polarity: 5.4) <methanol (polarity: 6.6) <water (polarity: 10.2; 50°C extraction was used in this layer), 200 ml of each extraction solvent was added. After 12 h, the extract was concentrated to approximately 1 ml using a rotary evaporator (RE-6000A). It was then fully dried in an evaporation dish inside a water bath at 20°C; the residue was dissolved in 1 ml of ethyl acetate, and distilled water was added to increase the volume to 50 ml (containing 2% ethyl acetate). This served as a stock solution of 1 g tissue (g ml^−1^) and was stored at 4°C for <5 days (Shao et al., 2016).

#### Evaluation of Germination Inhibition

During the end of November, the 1-g tissue (g ml^−1^) extracts of endocarps and embryos were diluted to 5, 10, 15, and 20% with distilled water. Next, 10 ml of extract or distilled water was added into sterile glass petri dishes (diameter, 9 cm; 3 filter paper were placed at the bottom). Distilled water containing 2% ethyl acetate served as the control. A total of 50 Chinese cabbage (*Brassica rapa* var. *glabra*) seeds (Variety Fengkang 90, Guanhe zhuangyuan Co., Ltd., China) were added to each plate replicate, and seeds were incubated at 25°C in the dark. Germination was monitored for 3 days until no further germination was noted; at this time, the length of the seedling hypocotyls and roots were measured using a vernier caliper. After selecting the extract with the strongest inhibitory effect, ryegrass (*Lolium perenne*) seeds (Big boss, Royal Barenbrug Group, Netherlands) were used to confirm the inhibitory effect of this extract following the same method. Three replicates of 50 seeds were used per treatment.

### Determination of Seed Extract Components

Gas chromatography (GC)-mass spectrometry (MS) was performed using HP6890/5975c GC–MS (Agilent, United States). For sample determination, 2 μl of the extract [ethyl acetate and methanol extracts of the endocarp, 1 g tissue (g ml^−1^)] was injected with a microsyringe. The chromatographic conditions were as follows: capillary column, hp-5 ms (60 m × 0.25 mm × 0.25 μm); initial temperature, 70°C (hold for 2 min), which was increased to 270°C at a rate of 5°C min^−1^ and then to 310°C at a rate of 8°C min^−1^ (hold for 9 min) for a total operation time of 56 min; vaporization chamber temperature, 250°C; carrier gas, high purity He (99.999%); pressure, 7.65 psi; carrier gas flow rate, 1.0 ml min^−1^; split ratio, 10:1; and solvent delay time, 6.0 min. The mass spectrum conditions were as follows: EI ion source with 230°C ion source temperature, 150°C quadrupole temperature, 70-EV electron energy, 34.6 μA emission current, 1,894 v multiplier voltage, 280°C interface temperature, and 29–450 amu mass range (Chen et al., 2010). Peaks in the total ion flow diagram were retrieved *via* the MS computer data system and were compared with the National Institute of Standards and Technology 2014 and Wiley 275 standard mass spectra to identify the volatile chemical components. The relative mass fraction of each chemical component was determined using the peak area normalization method.

### Seed Germination Test

#### Effects of Light and Temperature Treatments on Seed Germination

The *in situ* underground relative constant temperature, surface variable temperature, and light conditions were simulated. The light conditions were as follows: incubation in the dark (24 h) and dark/light (8/16 h), with 22.5 μmol m^−2^ s^−1^ light intensity and 40 ± 5% humidity. The constant treatment temperatures were 10°C, 20°C, 25°C, and 30°C. The alternating temperatures were 10/20°C, 20/25°C, and 25/30°C; the period of temperature change was 8/16 h. For each treatment, three replicates of 50 fresh seeds were used [fresh seeds were germinated in a sterile seed germination box (length 30 cm × width 20 cm × height 10 cm; 3 layers of filter paper were placed at the bottom) at 40 ± 5% humidity]. The seeds were observed every day, and the criterion for germination was radicle emergence (>2 mm). The experiment began in the early days of December 2017, and the treatment period was 240 days.

#### Effects of Hormone Treatment on Seed Germination

Fresh, intact seeds were soaked in GA_3_ aqueous solutions at concentrations of 100 mg L^−1^, 200 mg L^−1^, 400 mg L^−1^, 600 mg L^−1^, and 800 mg L^−1^ for 8 h. Afterward, the seeds were disinfected with 1% NaClO solution for 20 min at room temperature and then washed with sterile distilled water. Three replicates of 50 seeds were used per treatment. The seeds were incubated in germination boxes at 25°C in the dark for 30 days (length 30 cm × width 20 cm × height 10 cm); the conditions were dark incubation and 40 ± 5% humidity, and the treatment temperature was 25°C. The experiment began in the early days of December 2017, and the treatment period was 240 days.

#### Effects of Stratification Treatment on Seed Germination

The seeds were categorized into two groups of 50 seeds each and soaked in distilled water and 200 mg L^−1^ GA_3_ solution for 8 h. After disinfection with 1% NaClO solution for 20 min at room temperature, the seeds were washed with sterile distilled water. They were then mixed with wet sand (2 mm diameter; 30% humidity) in a ratio of 1:5 and divided into two equal parts, which were incubated at 4°C and 15°C for 240 days. Every 30 days, 150 seeds from each treatment (three replicates of 50 seeds) were assessed for germination in the dark at. The experiment began in the early days of December 2017, and the treatment period was 240 days.

### Data Analysis

Final germination percentage, germination energy, and germination index (GI) were calculated using the following formulas:


$$\begin{array}{l} \mathrm{Germination percentage}\;\left(\%\right)=\mathrm{number of germinated seeds}\\ \;/\mathrm{total number of seeds}\times 100\% \end{array}$$

$$\begin{array}{l} \mathrm{Germination energy}\;\left(\%\right)=\mathrm{number of germinated seeds when}\;\\ \mathrm{the germination percentage peaks}/\mathrm{total number of seeds}\times 100\% \end{array}$$

$$\mathrm{Germination index}\;\left(GI\right)=\sum Gt/Dt$$

where Gt is the number of germinated seeds per day and Dt is that of germination time (*d*).

The SPSS 21.0 statistical software package (IBM, Chicago, IL, United States) was used for data processing. One-way ANOVA and Duncan’s multiple comparison analysis were used to compare the treatment outcomes. All data were expressed as means ± standard deviations (SD). The data were inverse sine-transformed using the *asin()* function; data were expressed as means ± SDs and were analyzed using the SPSS 17.0 software (IBM, United States). Statistical differences among the treatments were analyzed using one-way ANOVA followed by Duncan’s *post-hoc* test. Statistical significance was set at *p* < 0.05. Origin 2019b (Origin Lab, Northampton, Ma, United States) and Adobe Illustrator 17.0 (California, ADBE, United States) software were used for drawing and processing.

## Results

### Characteristics of Seeds

**Figure 1:**
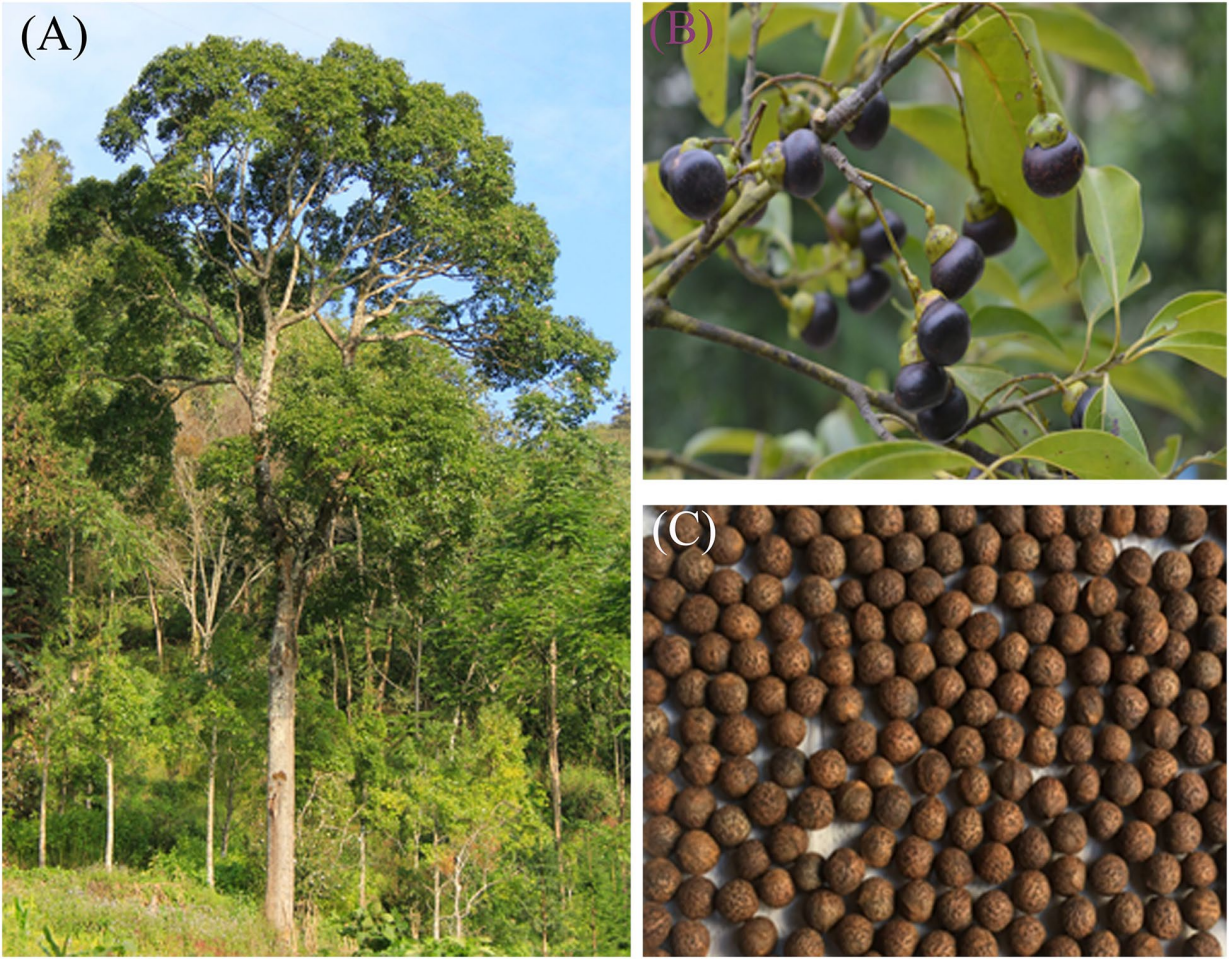
Morphology of *Cinnamomum migao* mature fruits and seeds. Mature *C. migao* tree **(A)**, fruits **(B)**, and dried seeds encapsulated by the endocarp **(C)**.

The mature *C. migao* fruits (Figure 1A) were baccate, 1.2–1.3 cm in diameter, and spherical with a slightly flat top (Figure 1B); these grew on the top of a goblet-shaped receptacle (Supplementary Figures S1A–D). The fresh seeds (encapsulated by the endocarp) were black and had a unique aroma. The seeds dried in the dark were brown and pea-shaped (Figure 1C).

The transverse and longitudinal axis diameters of the seeds were 10.73 ± 0.19 mm and 9.73 ± 0.09 mm, respectively, and the seed thickness was 9.31 ± 0.20 mm. The thousand-grain weight of the dry seeds was 421.34 ± 5.32 g, moisture content was 12.19 ± 1.21%, and viability was 98.67 ± 1.15%.

### Characteristics of the Endocarp and Embryo and Evaluation of Seed Imbibition

**Figure 2:**
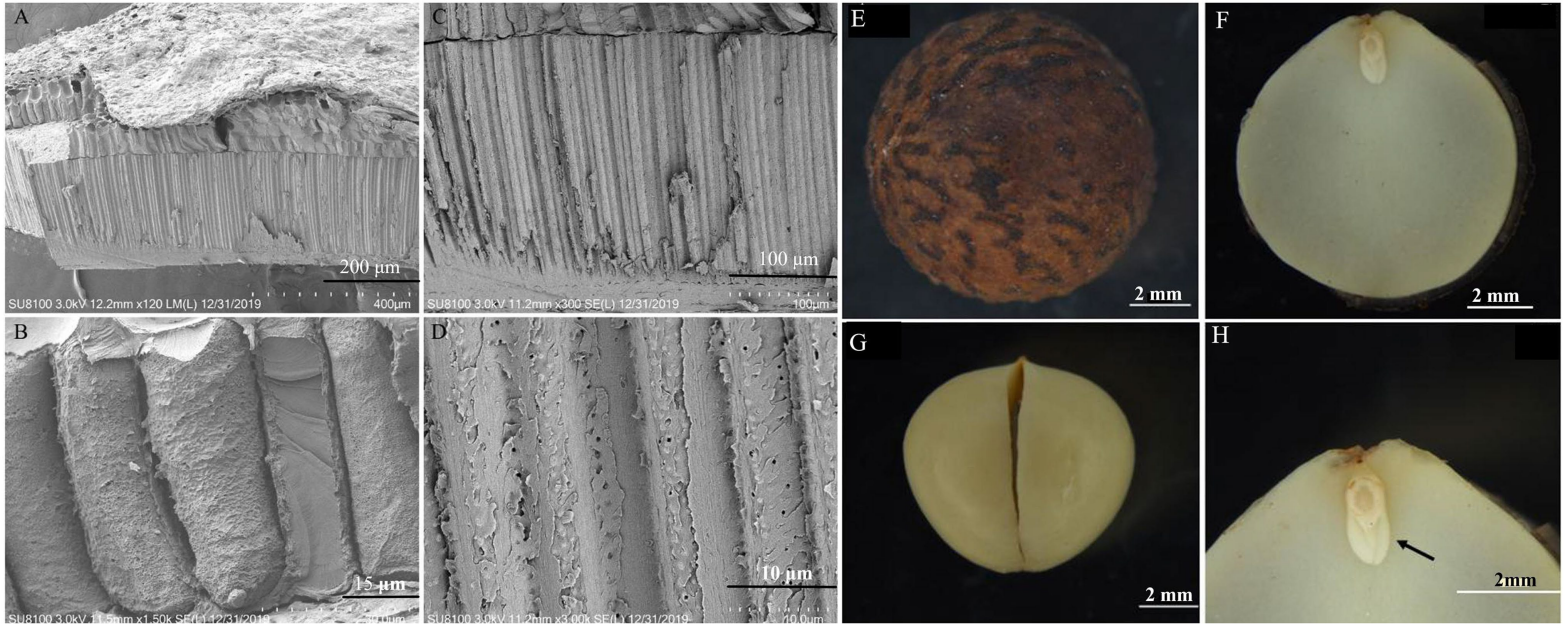
Morphology and anatomy of the *Cinnamomum migao* seed embryo and endocarp. Scanning electron microscope images of the endocarp **(A–D)**, scale bar: 2 mm. Light microscope images of the dry seed **(E)**, embryo **(F)**, cotyledon **(G)**, plumule (arrow), hypocotyl, and radicle **(H)**.

The endocarp was ossified and closely adhered to the testa, resulting in the separation of the testa from the embryo (Figure 2A). Inside the endocarp were two thick cotyledons (Figure 2B). The radicle, hypocotyl, and germ were small and cone-shaped, connecting the two cotyledons at the apex (Figure 2C). The obvious differentiation of the radicle, hypocotyl, and each part of the embryo indicates that these parts develop completely as the seeds mature (Figure 2D). The endocarp had a layer of stone cells (Figures 2E–H).

**Figure 3:**
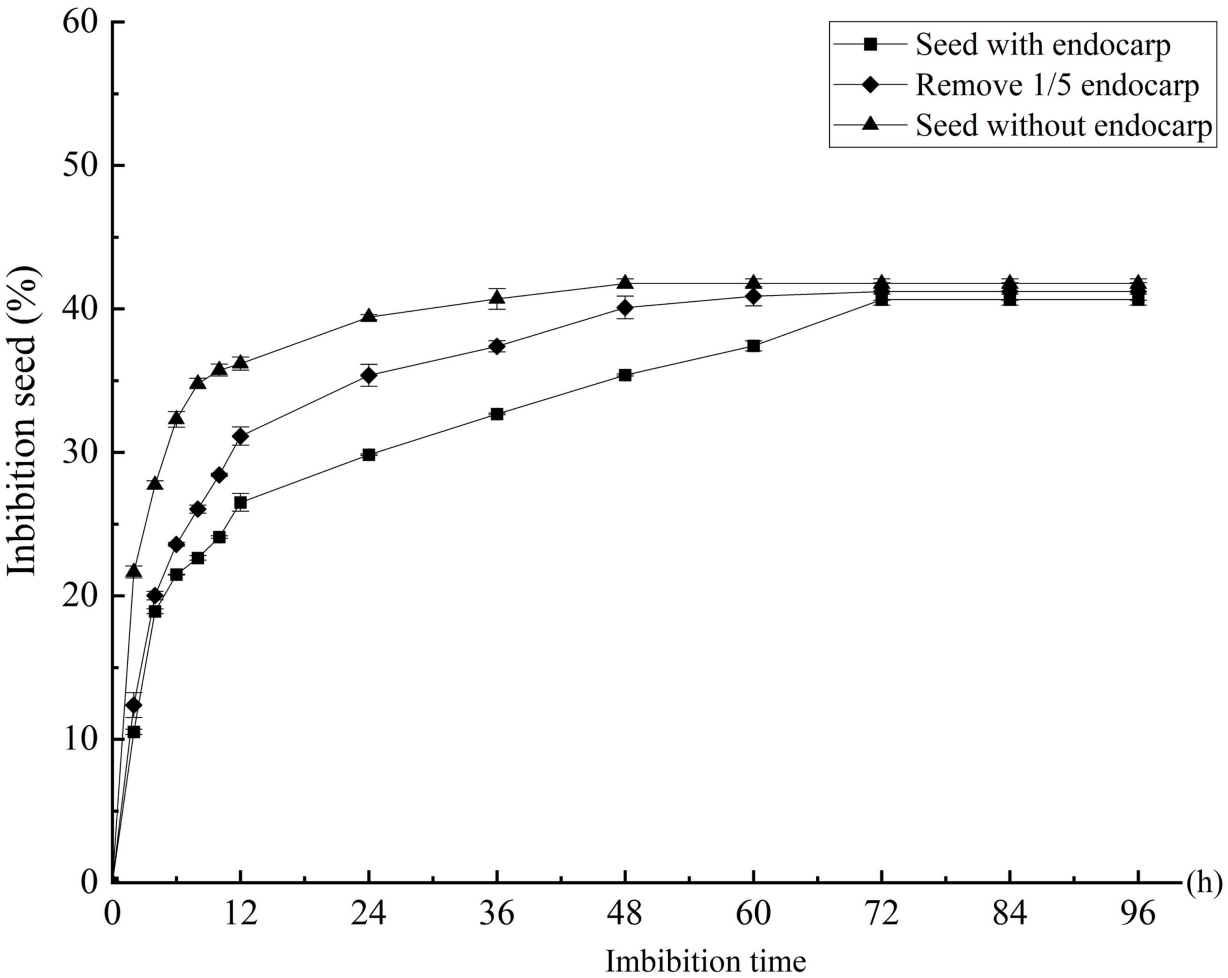
Effect of different treatments on the water absorption rate of *Cinnamomum migao* seeds. The *x*-axis denotes the water imbibition time of the seeds, and the *y*-axis denotes the water absorption percentage. Data are presented as means ± standard deviations for three replicates. Three replicates of 30 seeds each were used for each treatment.

The imbibition data of the *C. migao* seeds with the endocarp, with the partial endocarp, and without the endocarp are shown in Supplementary Figure S3. The seed weigh rapidly increased in the first 6 h. After 2 h of imbibition, the mass increases were 10.51, 12.38, and 21.65% for the seeds with the endocarp, with the partial endocarp, and without the endocarp, respectively; after 6 h, these were 21.47, 23.58, and 32.29%, respectively (Figure 3). Mass increase began to plateau after 12 h. The seeds with the endocarp, with the partial endocarp, and without the endocarp attained saturation after 72, 60, and 48 h of imbibition, respectively. Final mass increases were 40.64, 41.21, and 41.76%, respectively, with no significant differences across treatments (*p* > 0.05; Figure 3).

### Effects of Oxidant Treatment on Seed Dormancy and Germination

**Figure 4:**
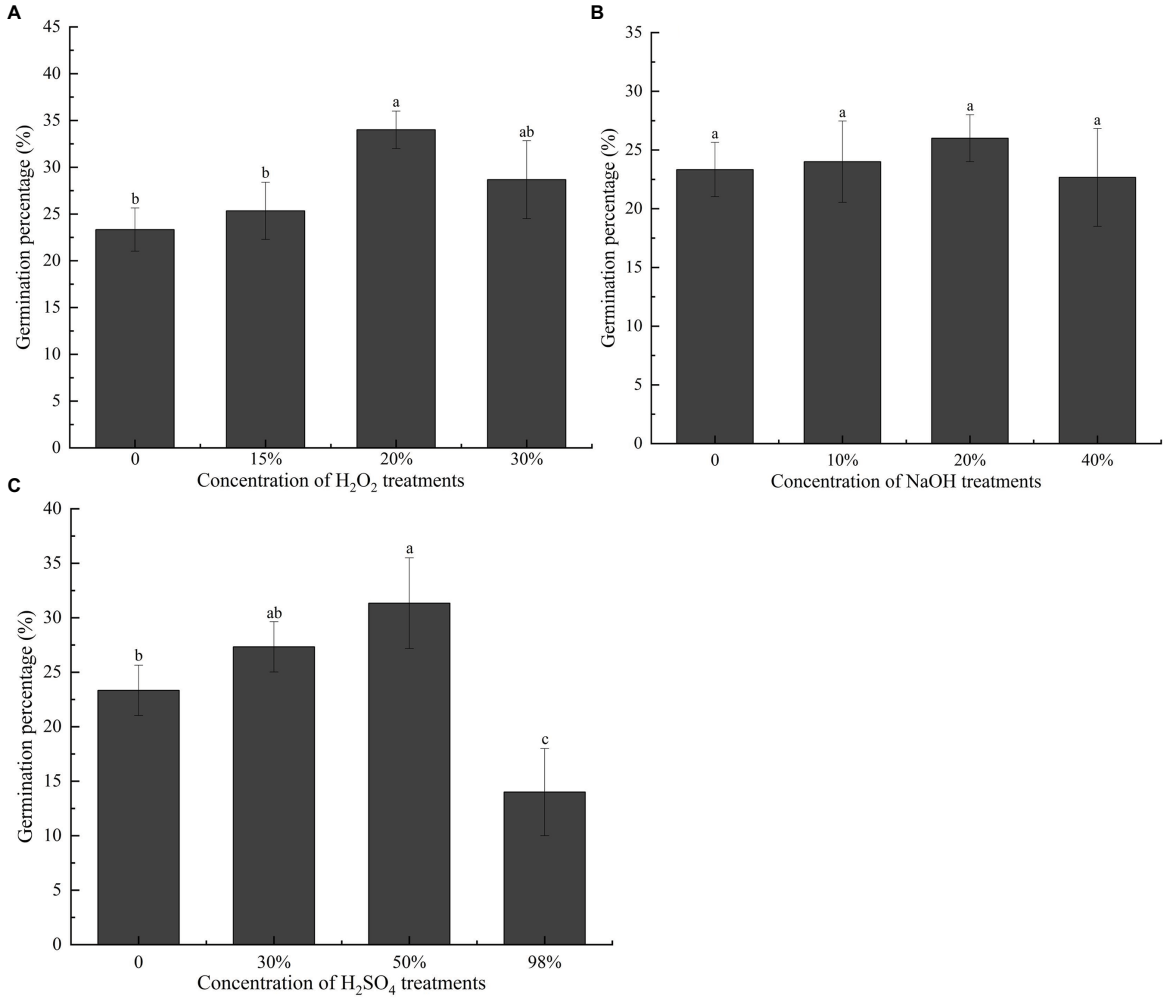
Effect of different chemical treatments on the final germination percentage of *Cinnamomum migao* seeds. Data are presented as means ± standard deviations (*n* = 3), and the values marked with different letters in various treatments vary significantly at *p* < 0.05. Three replicates of 50 seeds each were used for each treatment. **(A)** Different concentration of H_2_O_2_ treatment germination percentage of *C. migao* seed, **(B)** different concentration of NaOH treatment germination percentage of *C. migao* seed, **(C)** different concentration of H_2_SO_4_ treatment germination percentage of *C. migao* seed.

To further investigate how the endocarp influences germination, we treated the seeds with NaOH, H_2_SO_4_, and H_2_O_2_ before the germination test. Owing to corrosion or oxidation by chemical solvents, the endocarp thinned or the outer structure carbonized, blackened, and peeled off. The final germination percentage of seeds pretreated with 20% H_2_O_2_ was the highest at 34% (Figure 4A), which was significantly greater than that of the unstratified seeds (*p* < 0.05). The final germination percentages did not differ significantly (*p* > 0.05) in seeds pretreated with NaOH (Figure 4B). In seeds pretreated with H_2_SO_4_, the final germination percentage was 31.33% after pretreatment with 50% H_2_SO_4_ (Figure 4C), which was significantly higher than that of the unstratified seeds (*p* < 0.05). However, pretreatment with 98% H_2_SO_4_ significantly (*p* < 0.05) reduced the final germination percentage of the seeds.

### Inhibitory Effects of *C. migao* Seed Extract on Cabbage and Ryegrass Seed Germination

**Figure 5:**
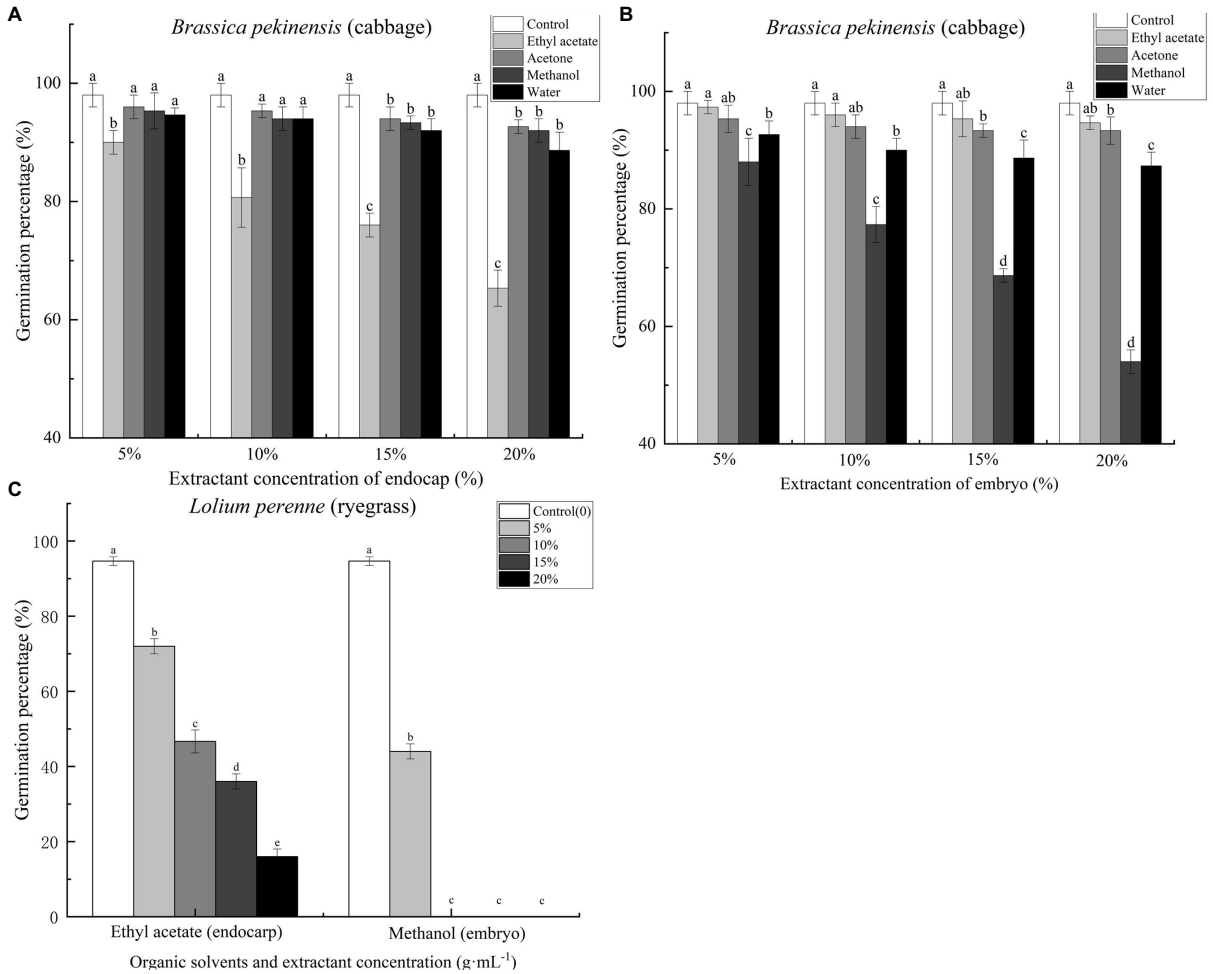
Inhibitory effects of different extracts of *Cinnamomum migao* seeds on the germination of cabbage and ryegrass seeds. **(A)** Endocarp extracts and **(B)** Embryo extracts. **(C)** Effect of endocarp and embryo extracts on ryegrass seed germination. The *x*-axis denotes the concentration of the extracts, and the *y*-axis denotes the germination rate. Data are presented as means ± standard deviations (*n* = 3). The values marked with different letters indicate the significance levels among the various extract treatments at the same concentration (*p* < 0.05). Three replicates of 50 seeds each were used for each treatment.

*Cinnamomum migao* endocarp and embryo extracts inhibited the germination of Chinese cabbage seeds (Supplementary Figure S3). Ethyl acetate endocarp extract, when applied at concentrations of 5, 10, 15, and 20%, significantly reduced the seed germination percentage of Chinese cabbage by 8.16, 17.69, 22.45, and 33.33%, respectively, compared with the control (Figure 5A). At the 5% concentration, acetone, methanol, and water extracts significantly (*p* < 0.05) inhibited the germination of cabbage seeds (Figure 5B). The methanol extract had the strongest inhibitory effect; concentrations of 5, 10, 15, and 20% reduced the final germination percentages of cabbage seeds by 10.20, 21.09, 29.93, and 44.90%, respectively, compared with the control (Figure 5B). Ryegrass seeds were treated with the *C. migao* extract showing the strongest inhibitory effects (endocarp: ethyl acetate extract; embryo: methanol extract) to confirm the inhibitory effects. The ryegrass seeds were significantly inhibited as the extract concentration increased (Figure 5C), and even the lowest concentration (5%) significantly reduced the final germination percentages. In addition, the growth of the cabbage and ryegrass seedlings was influenced by the *C. migao* seed extracts (Supplementary Tables S1–S3), with the ethyl acetate and methanol extracts of the endocarp and embryo, respectively, displaying the strongest inhibitory effects on the growth of cabbage seedlings.

### Evaluation of Endogenous Inhibitors in Seed Extract

**Figure 6:**
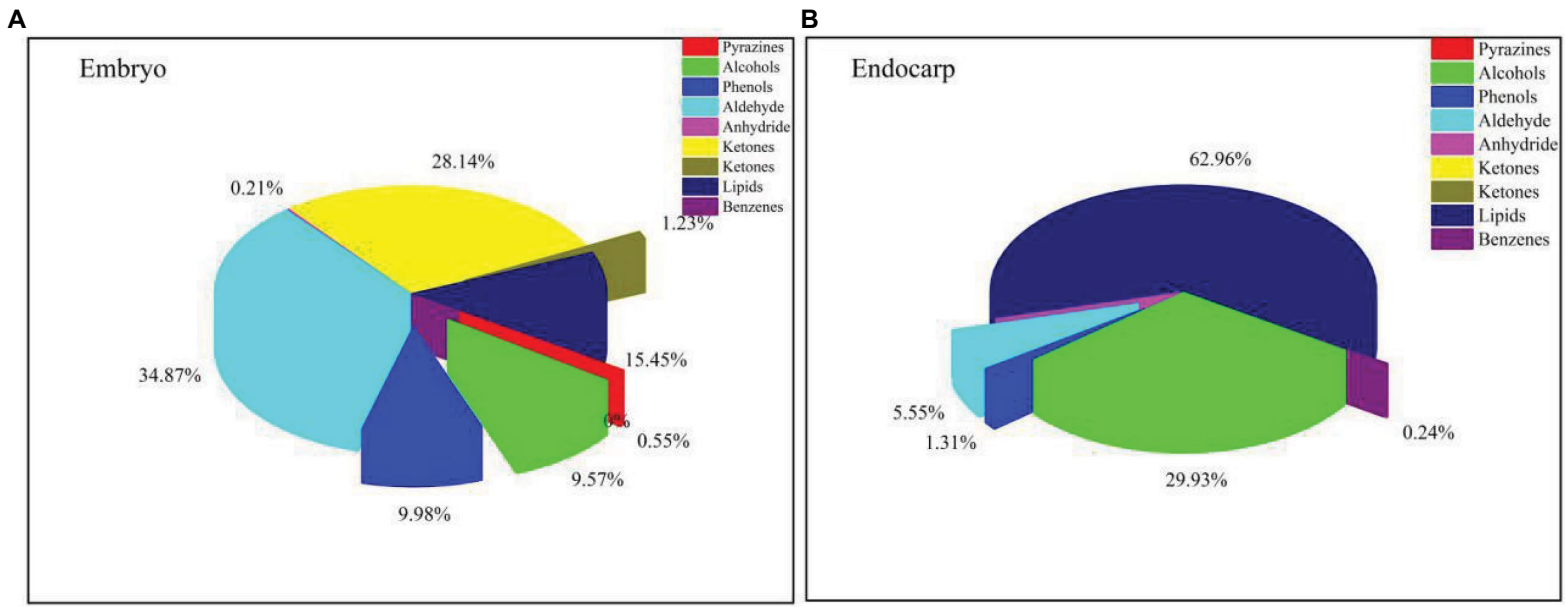
Types and relative percentages of the identified compounds in *Cinnamomum migao* fruits. **(A)**, Methanol extract of the *C. migao* embryo, concentration: 1 g tissue (g ml^−1^). **(B)**, Ethyl acetate extract of the *C. migao* endocarp, concentration: 1 g tissue (g ml^−1^).

The ethyl acetate and methanol extracts of *C. migao* seed endocarps were analyzed using GC-MS (Supplementary Figure S4). We identified 58 endogenous chemical compounds, including 25 in the ethyl acetate extract and 33 in the methanol extract. The primary components were as follows: 4 phenolic compounds, 11 aldehydes, 2 ethers, 8 ketones, 6 alcohols, 9 fatty acids, 11 lipids, 1 benzene, 1 pyrazine, 1 anhydride, and 1 alkene (Figure 6).

### Effects of Light and Temperature on Seed Germination

**Figure 7:**
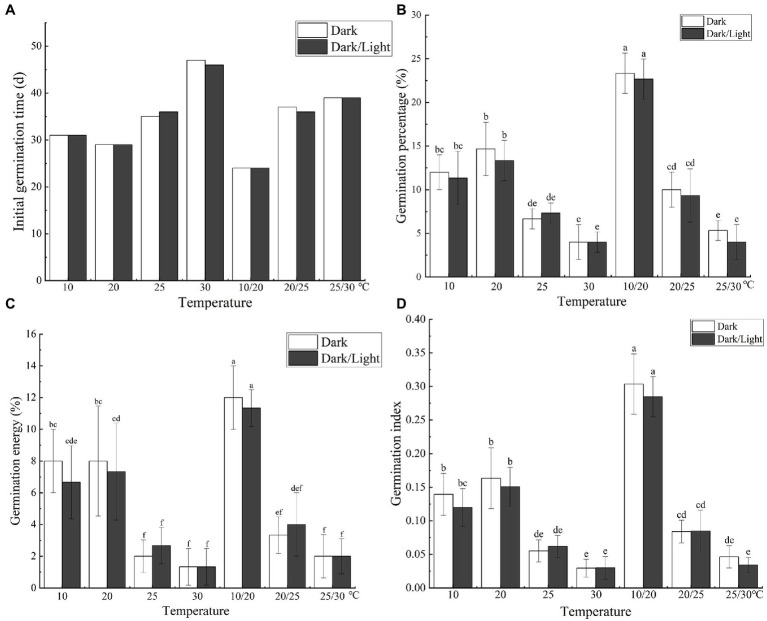
Effects of temperature and light treatments on the germination characteristics of *Cinnamomum migao* seeds. Data are presented as means ± standard deviations (*n* = 3), and the values marked with different letters in different treatments vary significantly (*p* < 0.05). Three replicates of 50 seeds were used in the germination test. **(A)** Initial germination time, **(B)** germination percentage, **(C)** germination energy, **(D)** germination index.

The temperature and light treatments influenced the initial seed germination and resulted in significant differences (*p* < 0.05) in the final germination percentage, germination vigor, and GI of *C. migao* seeds (Figure 7A). Seeds incubated at 30°C did not germinate until day 46, and the final germination percentage was the lowest at 4.0% (Figure 7B). ANOVA results (Supplementary Table S5) demonstrated that light (*F* = 0.706, *p* = 0.408) or the interaction between light and temperature (*F* = 0.157, *p* = 0.986) had no significant influence on germination. Nonetheless, the effect of temperature on the final germination percentage was significant (*F* = 55.33, *p* < 0.001). Thus, temperature is the primary factor in seed germination.

### Effects of Exogenous Hormone Treatment on Seed Germination

**Figure 8:**
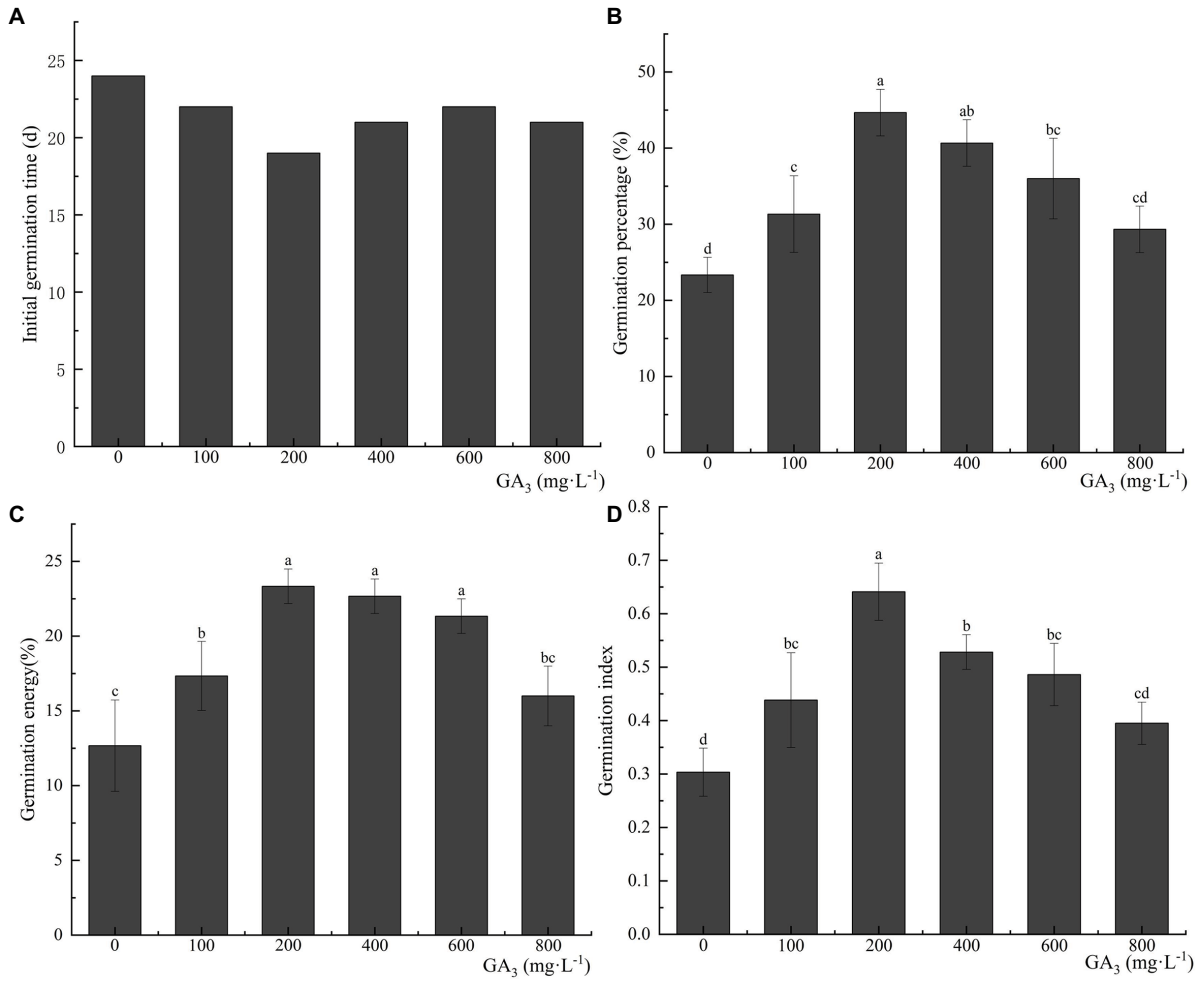
Effects of exogenous GA_3_ treatment on the germination characteristics of *Cinnamomum migao* seeds. Data are presented as means ± standard deviations (*n* = 3), and the values marked with different letters in different treatments vary significantly (*p* < 0.05). Three replicates of 50 seeds each were used in the germination test. **(A)** Initial germination time, **(B)** germination percentage, **(C)** germination energy, **(D)** germination index.

Pretreatment with GA_3_—a dormancy-releasing plant hormone—can effectively reduce the initial germination time of *C. migao* seeds. The final germination percentage, germination vigor, and GI improved with an increase in the GA_3_ concentration, reaching a peak at 200 mg L^−1^ and then decreasing (Figure 8). After the treatment with 200 mg L^−1^ GA_3_, the initial germination time was reduced to 19 days, which was 5 days less than that of the control seeds. The final seed germination percentage, germination vigor, and GI were 44.67 ± 3.06%, 23.33 ± 1.15%, and 0.64 ± 0.05 (representing growths of 91.47, 84.14, and 113.33%), respectively, compared with the control group (*p* < 0.05).

### Effects of Stratification Period on Seed Germination

**Figure 9:**
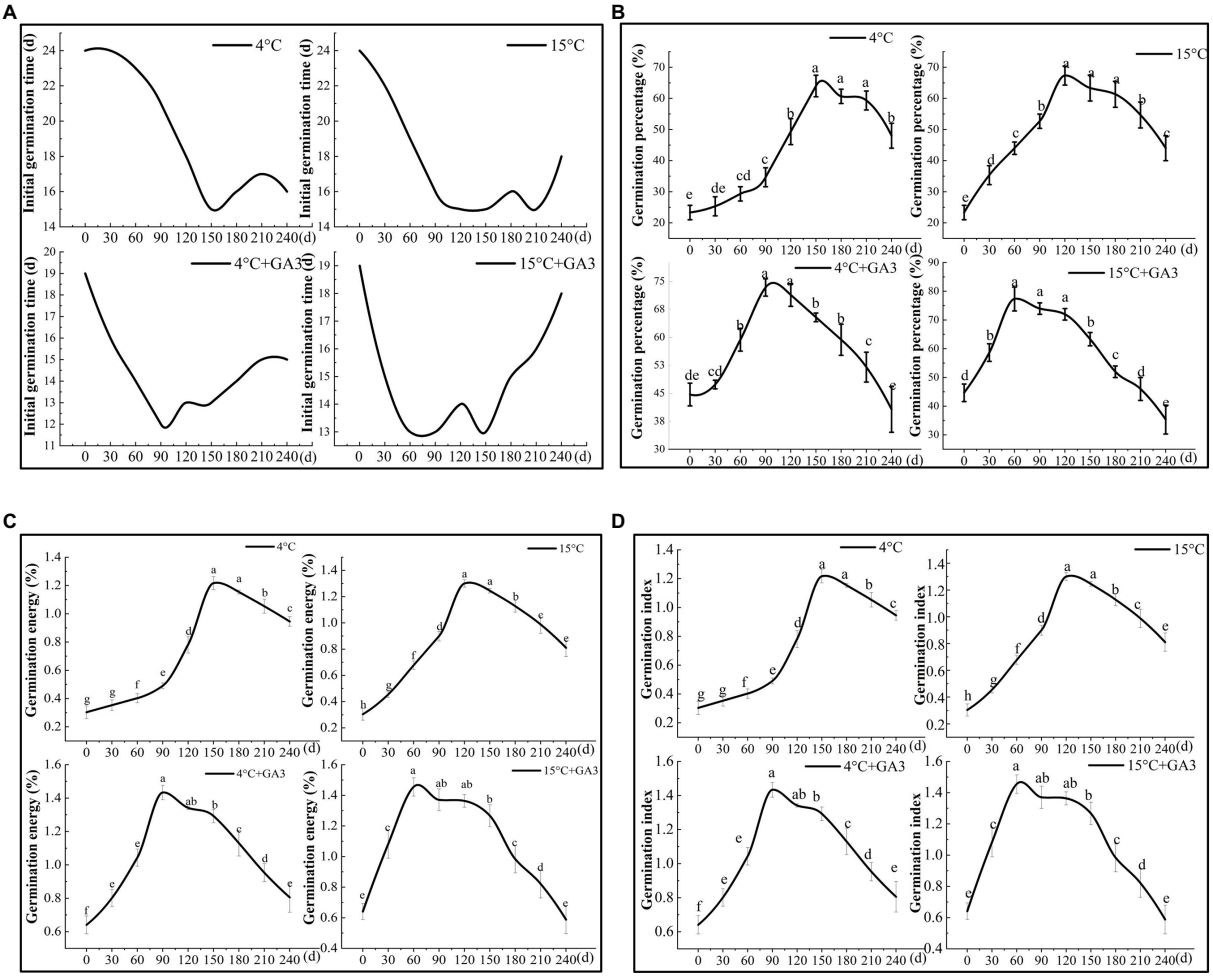
Effects of stratification on the germination characteristics of *Cinnamomum migao* seeds. Data are presented as means ± standard deviations (*n* = 3), and the values marked with different letters in different treatments vary significantly (*p* < 0.05). Three replicates of 50 seeds were used in the germination test. **(A)** Initial germination time, **(B)** germination percentage, **(C)** germination energy, **(D)** germination index.

The results of the stratification experiment revealed that the final germination percentage of *C. migao* seeds was higher under the cold/warm stratification + GA_3_ treatment than that under the stratification treatment alone (Figure 9). In addition, the germination percentage was higher in the warm-temperature stratification treatment (15°C) than that in the cold-temperature treatment (4°C). The germination ability of the seeds treated at 15 and 4°C with GA_3_ peaked at 60 (13 days for initial germination) and 90 (12 days for initial germination) days of stratification, respectively, which was significantly less than that of the unstratified seeds (Figure 9A). The final germination percentage, germination vigor, and GI were significantly higher (*p* < 0.05) in the 15°C + GA_3_- and 4°C + GA_3_-treatment groups than in the unstratified seeds by 73.11, 89.96, and 128.13% as well as 57.12, 82.90, and 123.44%, respectively (Figures 9B–D). The germination ability of seeds treated at 15 and 4°C peaked after 120 and 150 days of stratification, respectively. The initial germination time in both treatments was 15 days, which was 9 days less than that of the unstratified seeds. When 15°C + GA3 and 4°C + GA3 treatment compared with treatment in the absence of GA_3_, the final germination percentage, germination vigor, and GI of the unstratified seeds increased significantly by 188.60, 216.67, and 333.33% and 174.32, 194.63, and 306.67%, respectively (*p* < 0.05). After reaching the peak, the germination of the seeds under different treatments exhibited a downward trend with prolonged stratification time. The final germination percentage and vigor of seeds in 120–180 days declined in a nonsignificant manner (*p* > 0.05). In the stratification of 180–240 days, the seeds in each treatment group showed different degrees of reduction in the germination. Seeds in the 15°C + GA_3_ and 4°C + GA_3_ treatment groups had significantly reduced final germination percentages than those in the same temperature treatment groups but without GA_3_. Overall, stratification can effectively increase the final germination percentage of *C. migao* seeds, and GA_3_ treatment can significantly shorten the stratification period and can thus reduce the initial germination time. The warm-temperature (15°C) treatment was more effective in releasing the dormancy of the seeds than the low-temperature (4°C) treatment, and adding GA_3_ (15°C + GA_3_) to the warm-temperature stratification treatment further increased the final germination percentage.

## Discussion

### Dormancy Class and Potential Factors

The complex and diverse factors responsible for seed dormancy are associated with changes in the pericarp, seed coat, and embryo (Meyer, 2008). Therefore, we systematically investigated the possible causes of *C. migao* seed dormancy from the aspects of morphology, anatomy, and physiology.

MD refers to the dormancy due to the small embryo size, incomplete development, or lack of differentiation after seed maturation. The morphology of the seed embryo is generally spatulate, rudimentary, or linear; in some species, it occupies ≤1% of the seed volume (Jaganathan, 2020; Liu et al., 2020). For example, the *Lamprocapnos spectabilis* seed endosperm comprises the entire internal tissue of the seed, and the embryo is almost invisible (Cho et al., 2020). Similarly, the *Acer yangjuechi* seed embryo exhibits poor development and low vigor, which lead to a germination failure under suitable conditions (Chen et al., 2017). However, our results show that the mature embryos of *C. migao* seeds are fully developed, indicating the absence of MD (Figure 2; Supplementary Figure S1).

The endocarp of *C. migao* is water-permeable, and thus they do not have PY (Figure 2). Furthermore, no significant difference in the final percentages of imbibition was detected among the seeds with the endocarp, with the partial endocarp, and without the endocarp (Figure 3). The imbibition of *C. migao* seeds was similar to that of *Phoebe bournei* seeds belonging to the same family, in which the pericarp does not hinder imbibition (Li and Min, 2020). *C. migao* seeds with the partial endocarp absorbed water faster than those with the intact endocarp; seeds without the endocarp absorbed water even faster than those with the partial endocarp. This finding is similar the results obtained for the endocarp of *Prunus mahaleb* (Murphy et al., 2010) and *Phillyrea angustifolia* (Mira et al., 2015). However, in the present study, the intact seeds were capable of absorbing the same amount of water as that of the seeds without the endocarp, although the imbibition was slower. Hence, the endocarp was not impermeable, which eliminated the PY of *C. migao* (Figure 3).

To assess PD, we examined whether the *C. migao* endocarp and embryo extracts contain compounds that inhibit the germination of cabbage and ryegrass seeds. Specifically, the ethyl acetate extract of the endocarp and the methanol extract of the embryo showed significant inhibitory effects. The methanol extract completely inhibited ryegrass germination at concentrations of >0.05 g ml^−1^ (Figure 5B). The primary components of the extracts with the strongest inhibitory effects were lipids, fatty acids, ketones, aldehydes, and phenolic compounds. Previous studies have demonstrated that inhibitory compounds in the endocarp or the hard middle layer of the testa contribute to low and unstable seed germination (Hamilton and Carpenter, 1976; Richmond and Ghisalberti, 1994; Wu and Shen, 2021). Phenolic and lipid compounds present in the seeds have been widely shown to induce seed dormancy and prevent germination (Graeber et al., 2012; Inácio et al., 2013; Smýkal et al., 2014). Inhibitory compounds present in the seeds can cause physiological inhibition by hindering the growth potential or breakthrough ability of the embryo; as a result, it fails to break the mechanical resistance of the endocarp, thereby negatively influencing seed germination (Baskin and Baskin, 2014). Similarly, previous studies have reported that phenolic compounds present in *Lycopersicon esculentum* (tomato), *Fagus sylvatica*, and *Pinus laricio* in the seeds inhibit the metabolism of key enzymes involved in seed germination (Muscolo et al., 2001; El-Araby et al., 2006). Therefore, according to the classification system of Baskin and Baskin (2004), we determined that *C. migao* seed dormancy is physiological in nature and is due to endogenous inhibitors and low growth potential of the embryo.

### Effects of Various Treatments on *C. migao* Seed Dormancy

For seeds with obvious dormancy, its release involves changes in the external environment and the physiology and biochemistry of the seed. The release of seed dormancy primarily occurs through the regulation of light, temperature, hormones, and stratification (Cosmas et al., 2019; Hou et al., 2019; Yang et al., 2019). Therefore, we evaluated the effects of light, temperature, an exogenous hormone, and stratification on *C. migao* seed dormancy and germination.

No significant differences in dormancy release were observed in the seeds under the same temperature but different light treatments. Light neither reduced the initial germination time nor influence the final germination percentage, germination vigor, or GI (Supplementary Table S5). Light-requiring seeds are generally small (Koutsovoulou et al., 2014; Yang et al., 2020), which allows the external light to be perceived by the seed pigments, integrating the metabolism and signal transduction of abscisic acid and GA to control seed dormancy and germination (Bentsink and Koornneef, 2008; Shu et al., 2016). However, *C. migao* seeds are relatively large and are encapsulated by the firm endocarp (Figures 1, 2), which prevents the internal pigments from perceiving external light (Liu and Lin, 1995).

The low constant temperature and alternating temperatures reduced the initial germination time and significantly increased the final germination percentage, germination vigor, and GI, with the treatment effect being the strongest at 10/20°C. Compared with the interaction of light and temperature, temperature alone significantly promoted the release of dormancy (Figure 7). The natural distribution of *C. migao* is limited to dry and hot valleys, and the 10/20°C alternating temperature condition is similar to the habitat temperature in spring (Tian et al., 2015). The changing temperature stimulates enzymatic activities in these seeds, which in turn promotes the transformation of the stored materials and releases the dormancy. The 10/20°C alternating temperature treatment may be in line with the natural temperature change in the environment (Chahtane et al., 2016; Kildisheva et al., 2019).

The role of the exogenous hormone GA_3_ in releasing seed dormancy and increasing the final germination percentage has been extensively studied (Linkies and Leubner-Metzger, 2012). GA_3_ treatment has been demonstrated to effectively enhance the final germination percentage of *Buglossoides arvensis* and *Cyclocarya paliurus* seeds (Fang et al., 2006; de Las Mercedes et al., 2021). GA_3_ improves α-amylase hydrolysis and glyoxylate cycle enzymatic activities in seeds, thereby activating energy metabolism and enhancing the growth potential of the embryo (Song et al., 2019). In addition, the cold/warm stratification treatment has been shown to release PY (Wolkis et al., 2020). Similar to physical polishing, stratification increases the growth potential of the embryo, thus allowing it to overcome the mechanical resistance to its growth (Baskin and Baskin, 2014). Our results showed that seed treatment with 200 mg L^−1^ pure exogenous GA_3_ significantly reduces the initial germination time and the final germination percentage (Figure 8). The 15°C stratification was significantly better than the 4°C stratification, and stratification after GA_3_ pretreatment resulted in earlier germination peaks than those observed with stratification alone. The combination of 15°C + GA_3_ was particularly effective, inducing initial germination 6 days earlier than the control seeds. The final germination percentage was as high as 77.33 ± 4.16%, which is significantly higher than those noted in the alternating temperature, light, single hormone, and single stratification treatments (Figure 9). Therefore, combining GA_3_ pretreatment with stratification was most effective in the rapid release of *C. migao* seed dormancy (Figure 9B). The hormone treatment activates and accelerates the metabolic activities of the seeds, promoting cell division and radicle growth (Yang et al., 2019), and the stratification treatment influences the hormone contents and metabolic processes. The combination of exogenous hormones and stratification treatment thus accelerated the physiological and biochemical processes.

### Release of Seed Dormancy and Natural Regeneration of the *C. migao* Population

Temperature and cold stratification treatment were effective in releasing the dormancy of *C. migao* seeds (Figure 9); however, light and dark treatments had no significant effect on dormancy release (Figure 7). The habitats of *C. migao*—the forests—are heavily impacted by human activities, including fruit harvesting as well as understorey vegetation and leaf litter removal. Therefore, although a large number of fruits fall on the ground after ripening, these are not covered by litter (Zhou, 2003). Owing to the relatively dry surface soil conditions in winter, *C. migao* seeds may be unable to absorb sufficient water for effective alternating temperature stratification. This phenomenon has been observed in several species. Exposure to the soil surface increases the risk of animal (e.g., rodents) foraging in winter (Gao et al., 2021). In fact, we noted several *C. migao* seeds that were apparently chewed by rodents. Therefore, the lack of an effective stratification treatment because of human disturbance may significantly hinder the release of dormancy and lead to an extreme shortage of *C. migao* seedlings. The results provide important insights into the successful regeneration of the *C. migao* population. For example, covering the seeds under the litter to ensure effective stratification in winter will facilitate seed germination. On the other hand, collecting seeds to artificially release seed dormancy can prevent animal foraging (Chen et al., 2020). Furthermore, the reintroduction of *C. migao* seedlings into wild population should be considered an effective method to increase the population of this arbor (Wright et al., 2009).

## Conclusion

*Cinnamomum migao* seeds absorb water easily, and the embryo is well developed; no obvious mechanical hindrance to the germination process was identified. However, the seed embryo and endocarp were rich in phenols, aldehydes, which reduced the final germination percentages of cabbage and ryegrass seeds. These compounds may inhibit the germination of fresh *C. migao* seeds and thus induce/enhance dormancy. The abovementioned results suggest that *C. migao* seeds exhibit PD. Our findings demonstrated that seed dormancy release was not influenced by light; however, an exogenous hormone, temperature, and stratification treatments influenced seed dormancy release. Overall, GA_3_ pretreatment followed by a 15°C stratification treatment for 60 days was most effective in relieving *C. migao* seed dormancy.

## Data Availability Statement

The raw data supporting the conclusions of this article will be made available by the authors, without undue reservation.

## Author Contributions

X-lH and X-fL: conceptualization and data curation. J-zC: writing–original draft preparation. Q-wS and J-mL: methodology. X-fX, LP, and LZ: writing–review and editing. All authors have read and approved the final version of the manuscript.

## Funding

This work was supported by the National Natural Science Foundation of Unite Project, China (grant number: U1812403-2) and the Guizhou Science and Technology Program [Qiankehe (2019) 2774].

## Conflict of Interest

The authors declare that the research was conducted in the absence of any commercial or financial relationships that could be construed as a potential conflict of interest.

## Publisher’s Note

All claims expressed in this article are solely those of the authors and do not necessarily represent those of their affiliated organizations, or those of the publisher, the editors and the reviewers. Any product that may be evaluated in this article, or claim that may be made by its manufacturer, is not guaranteed or endorsed by the publisher.
