# Supplementary material for: Seed Dormancy Release and Germination Requirements of Cinnamomum migao, an Endangered and Rare Woody Plant in Southwest China
Source: Front Plant Sci. 2022 Jan 27;13:770940. doi: 10.3389/fpls.2022.770940 (PMC8828499; doi:10.3389/fpls.2022.770940)
Supplement: Supplementary file 1 [file Data_Sheet_1.docx]

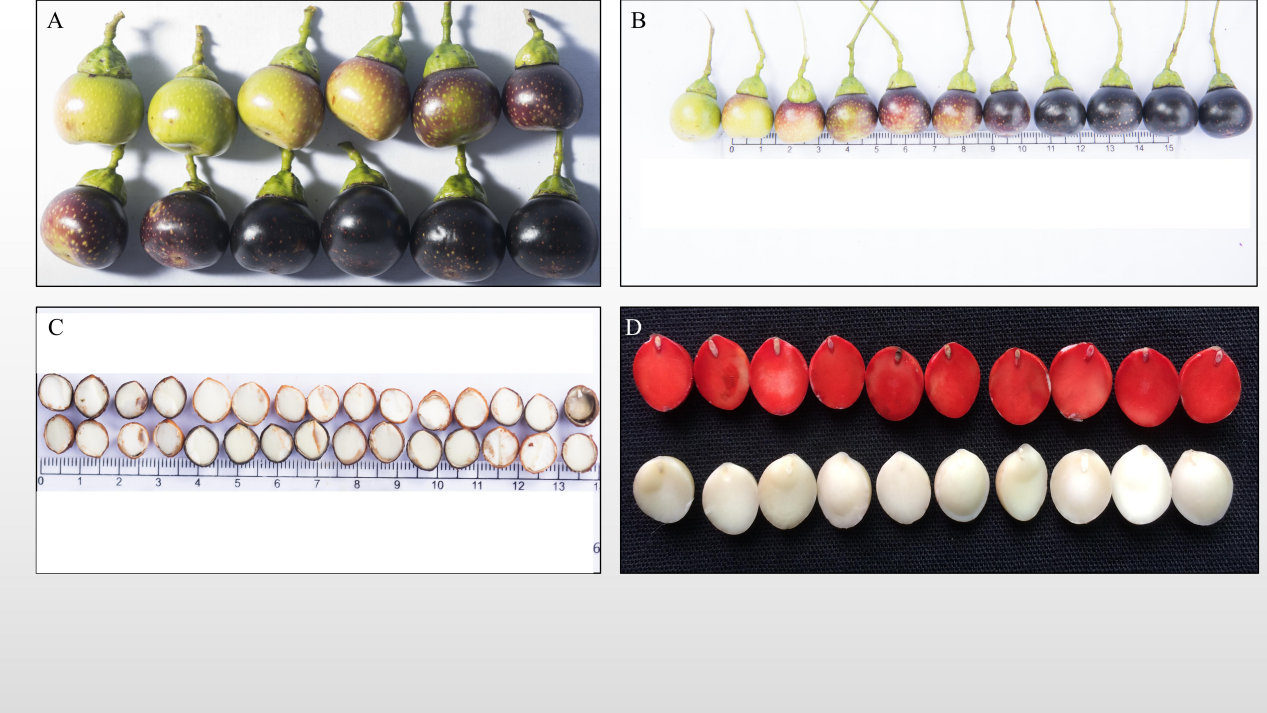


**Fig. S1 Fresh fruit of *Cinnamomum migao,* and viabilible test results of seeds** **of *Cinnamomum migao*;** A-B, Fruits of *C. migao*, the ripen fruit of *C. migao* were black. In all experiments, only total black ripen fruits was selected, and other colour gradient fruit (unripe seeds) was not selected at experiment; C, Embryo of *C. migao* without endocarp and testa. D, *C. migao* seeds could be dyed red by TTC indicates had good activity, boiled seeds were used as negative control (boiling water for 20 min) could not be dyed red by TTC.


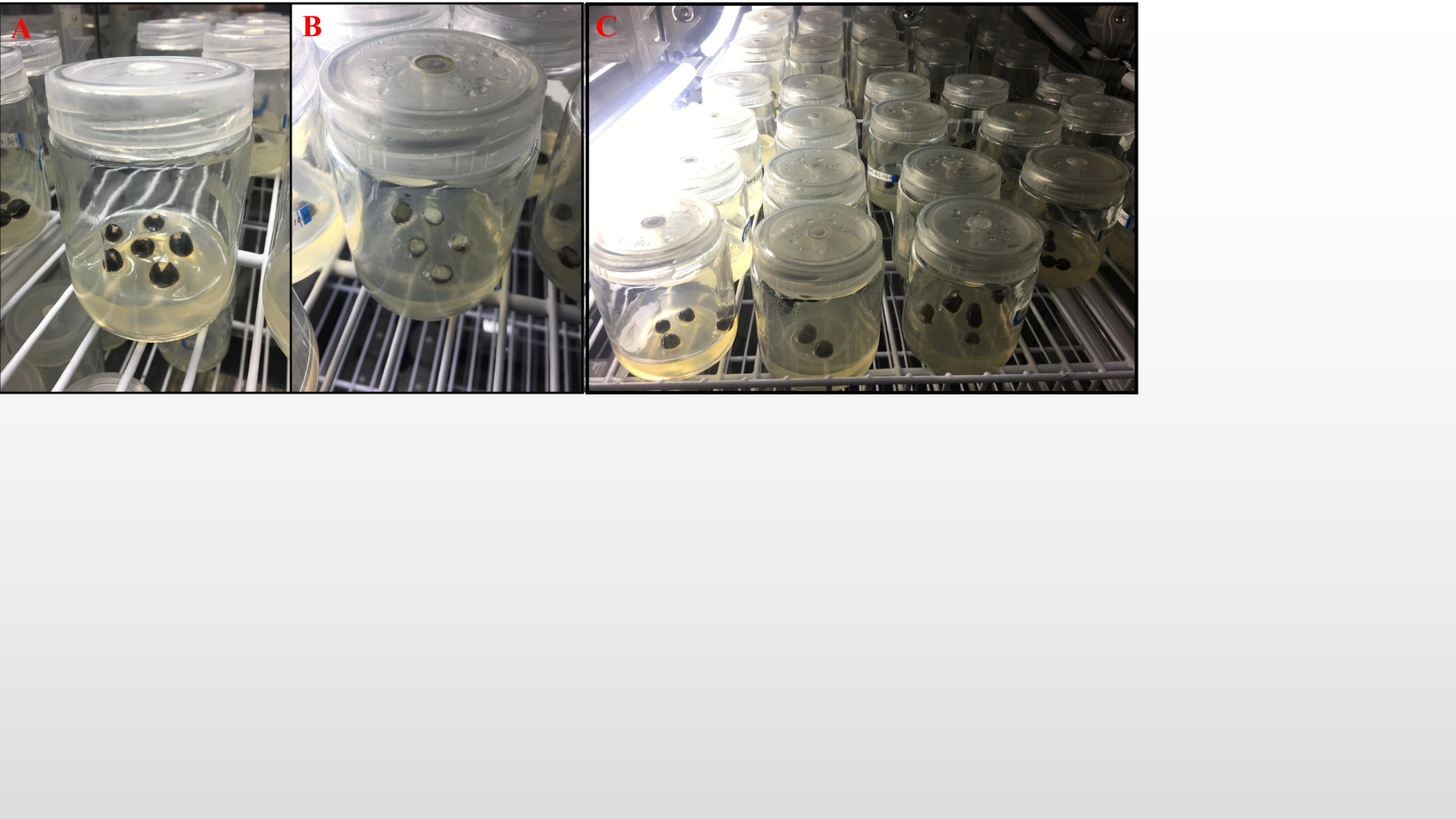


**Fig. S2 Different physical scratch modes of *Cinnamomum migao* seeds.** A, Seeds with 1/5 endocarp removed. B, Seeds without endocarps. C, Seeds with endocarps.


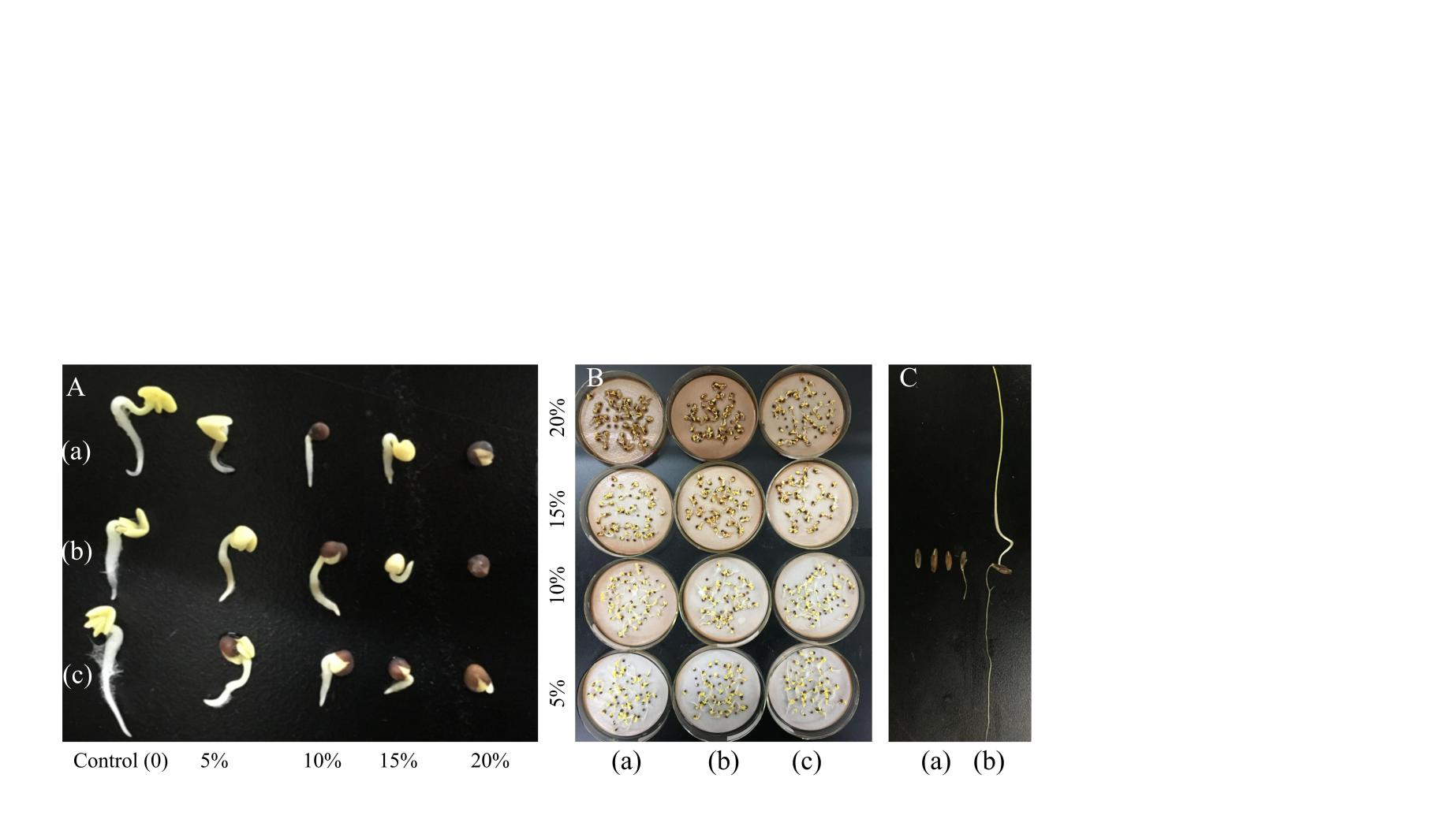


a

a

b

**Fig. S3 Effects of endogenous inhibitors of *Cinnamomum migao* seeds on Chinese cabbage and ryegrass germination of seeds**; A, The inhibition effect of cabbage seeds increased gradually from left to right (0%, 5%, 10%, 15%, 20%). (a), Endocarp (ethyl acetate), (b), Embryo (methanol), (c), Embryo (water); B, Inhibition test for Chinese cabbage. The inhibition effect of cabbage seeds increased gradually from bottom to top (5%, 10%, 15%, 20%). (a), Endocarp (ethyl acetate) (b), Embryo (methanol), (c), Embryo (water); C, (a), Concentration of methanol extract from *C. migao* seeds was 5%, the germination of ryegrass seeds was significantly inhibited (*p* < 0.05), (b), water was negative control. When final germination rate was recorded at the end of the germination experiment (6 days).

**Fig. S4 TIC diagram of GC-MS analysis of ethyl acetate and methanol extraction of *Cinnamomum migao* seed**

**Table S1 Inhibition effect of extracts from the endocarp of *Cinnamomum migao* seed on the growth of cabbage seedlings**

| **Concentration**  **(%)** | **Aqueous extracts** | **Hypocotyl length**  **(mm)** | **Inhibitory activity**  **(%)** | **Root length**  **(mm)** | **Inhibitory activity**  **(%)** |
| --- | --- | --- | --- | --- | --- |
| 5 | Control | 3.40 ± 0.11a | 0 | 11.75 ± 0.25a | 0 |
|  | Ethyl acetale | 2.86 ± 0.23b | 15.64 | 10.43 ± 0.20c | 11.29 |
|  | Acetone | 3.31 ± 0.17a | 2.59 | 11.57 ± 0.27ab | 1.55 |
|  | Methanol | 3.28 ± 0.14a | 3.55 | 11.44 ± 0.31b | 2.66 |
|  | Distilled water | 3.33 ± 0.20a | 1.80 | 11.62 ± 0.45ab | 1.16 |
| 10 | Control | 3.40 ± 0.11a | 0 | 11.75 ± 0.25a | 0 |
|  | Ethyl acetale | 2.79 ± 0.12d | 17.83 | 9.37 ± 0.31d | 20.25 |
|  | Acetone | 3.27 ± 0.14b | 3.63 | 11.26 ± 0.27c | 4.20 |
|  | Methanol | 3.11 ± 0.18c | 8.41 | 11.21 ± 0.32c | 4.64 |
|  | Distilled water | 3.29 ± 0.15b | 3.03 | 11.46 ± 0.31b | 2.51 |
| 15 | Control | 3.40 ± 0.11a | 0 | 11.75 ± 0.25a | 0 |
|  | Ethyl acetale | 2.28 ± 0.17d | 32.87 | 8.20 ± 0.33d | 30.18 |
|  | Acetone | 3.12 ± 0.12b | 8.00 | 11.19 ± 0.25b | 4.76 |
|  | Methanol | 2.94 ± 0.19c | 13.37 | 10.97 ± 0.34c | 6.68 |
|  | Distilled water | 3.21 ± 0.18b | 5.35 | 11.28 ± 0.22b | 4.03 |
| 20 | Control | 3.40 ± 0.11a | 0 | 11.75 ± 0.25a | 0 |
|  | Ethyl acetale | 2.11 ± 0.14e | 37.76 | 6.50 ± 0.12d | 44.66 |
|  | Acetone | 2.93 ± 0.15c | 13.76 | 10.85 ± 0.30b | 7.68 |
|  | Methanol | 2.73 ± 0.16d | 19.65 | 10.75 ± 0.31c | 8.53 |
|  | Distilled water | 3.07 ± 0.15b | 9.47 | 10.93 ± 0.29b | 6.98 |

Values are expressed as means ± SD for three replicates, and the values marked with different letters in same columns were significantly different at *P* < 0.05.

**Table S2 Inhibition effect of extracts from the embryo of *Cinnamomum migao* seed on the growth of cabbage seedlings.** Values are expressed as means ± SD (n = 3) for three replicates, and the values marked with different letters in same columns were significantly different at *P* < 0.05.

| **Concentration**  **(%)** | **Aqueous extracts** | **Hypocotyl length**  **(mm)** | **Inhibitory activity**  **(%)** | **Root length**  **(mm)** | **Inhibitory activity**  **(%)** |
| --- | --- | --- | --- | --- | --- |
| 5 | Control | 3.40 ± 0.11a | 0 | 11.75 ± 0.25a | 0 |
|  | Ethyl acetale | 3.30 ± 0.11b | 2.68 | 11.52 ± 0.36b | 1.97 |
|  | Acetone | 3.21 ± 0.09c | 5.51 | 11.46 ± 0.28b | 2.45 |
|  | Methanol | 2.79 ± 0.17d | 17.77 | 10.38 ± 0.27d | 11.66 |
|  | Distilled water | 3.19 ± 0.16c | 6.00 | 11.15 ± 0.27c | 5.10 |
| 10 | Control | 3.40 ± 0.11a | 0 | 11.75 ± 0.25a | 0 |
|  | Ethyl acetale | 3.14 ± 0.12b | 7.43 | 11.37 ± 0.25b | 3.23 |
|  | Acetone | 3.08 ± 0.09b | 9.31 | 11.28 ± 0.23b | 4.03 |
|  | Methanol | 2.47 ± 0.11c | 27.41 | 9.24 ± 0.35d | 21.34 |
|  | Distilled water | 3.12 ± 0.18b | 8.13 | 10.92 ± 0.33c | 7.06 |
| 15 | Control | 3.40 ± 0.11a | 0 | 11.75 ± 0.25a | 0 |
|  | Ethyl acetale | 3.09 ± 0.11b | 8.98 | 11.13 ± 0.27b | 5.27 |
|  | Acetone | 2.97 ± 0.15c | 12.42 | 10.93 ± 0.38c | 7.03 |
|  | Methanol | 2.13 ± 0.16d | 37.16 | 8.07 ± 0.19e | 31.35 |
|  | Distilled water | 2.94 ± 0.17c | 13.45 | 10.67 ± 0.28d | 9.20 |
| 20 | Control | 3.40 ± 0.11a | 0 | 11.75 ± 0.25a | 0 |
|  | Ethyl acetale | 2.95 ± 0.19b | 13.12 | 10.99 ± 0.34b | 6.51 |
|  | Acetone | 2.84 ± 0.20c | 16.43 | 10.22 ± 0.27c | 13.03 |
|  | Methanol | 1.75 ± 0.07e | 48.59 | 5.62 ± 0.33e | 52.16 |
|  | Distilled water | 2.56 ± 0.0.16d | 24.62 | 9.54 ± 0.0.27d | 18.79 |

**Table S3 Inhibition effect of extracts from the endocarp and embryo of *Cinnamomum migao* seed on the growth of *Lolium perenne* seedlings.** Values are expressed as means ± SD (n = 3) for three replicates, and the values marked with different letters in same columns were significantly different at *P* < 0.05.

| **Concentration**  **(%)** | **Aqueous extracts** | **Hypocotyl length**  **(mm)** | **Inhibitory activity**  **(%)** | **Root length**  **(mm)** | **Inhibitory activity**  **(%)** |
| --- | --- | --- | --- | --- | --- |
| Ethyl acetale | Control | 58.68 ± 2.79a | 0 | 64.72 ± 3.72a | 0 |
|  | 5 | 49.16 ±2.74b | 16.23 | 49.75 ± 2.55b | 15.21 |
|  | 10 | 34.21 ± 1.95c | 41.70 | 36.05 ± 1.89c | 38.56 |
|  | 15 | 23.34 ± 2.94d | 60.23 | 26.17 ± 1.41d | 55.40 |
|  | 20 | 13.12 ± 1.31e | 77.64 | 14.03 ± 1.64e | 76.08 |
| Methanol | Control | 58.68 ± 2.79a | 0 | 64.72 ± 3.72a | 0 |
|  | 5 | 12.52 ± 1.51b | 78.66 | 12.83 ± 1.26b | 78.14 |
|  | 10 | 0 | 100 | 0 | 100 |
|  | 15 | 0 | 100 | 0 | 100 |
|  | 20 | 0 | 100 | 0 | 100 |

**Table S4 Components of inhibitive substances by analysis of GC-MS in ethyl acetate and methanol extracts of *Cinnamomum migao* seed.**

| **Chemical reagents** | **Serial number** | **Time (min)** | **Chemical compound** | **Molecular formula** | **Molecular weight** | **Relative content (%)** |
| --- | --- | --- | --- | --- | --- | --- |
| **Ethyl acetale**  (**Endocarp)** | 1 | 9.847 | Nonanal | C_9_H_18_O | 142 | 0.078 |
|  | 2 | 10.479 | Glycerol | C_3_H_8_O_3_ | 92 | 0.247 |
|  | 3 | 12.411 | p-Cymene | C_10_H_14_ | 134 | 0.020 |
|  | 4 | 12.674 | 1,8-Cineole | C_10_H_18_O | 154 | 0.046 |
|  | 5 | 14.201 | Diacetylglycerol | C_7_H_12_O_5_ | 176 | 3.347 |
|  | 6 | 16.227 | Octanoic acid | C_8_H_16_O_2_ | 144 | 0.206 |
|  | 7 | 17.095 | p-Cymen-8-ol | C_10_H_14_O | 150 | 0.062 |
|  | 8 | 17.274 | 4-Isopropyl-2-cyclohexen-1-one (cryptone) | C_9_H_14_O | 138 | 0.054 |
|  | 9 | 18.244 | 2-Hydroxy-1,8-cineole | C_10_H_18_O_2_ | 170 | 0.129 |
|  | 10 | 18.470 | 1,2-Diacetylglycerol | C_7_H_12_O_5_ | 176 | 2.184 |
|  | 11 | 19.988 | 2-Undecanone | C_11_H_22_O | 170 | 0.026 |
|  | 12 | 20.242 | Carvacrol | C_10_H_14_O | 150 | 0.029 |
|  | 13 | 20.779 | Methyl caprate | C_11_H_22_O_2_ | 186 | 0.069 |
|  | 14 | 21.345 | Glycerin triacetate | C_9_H_14_O_6_ | 218 | 0.063 |
|  | 15 | 22.287 | Decanoic acid | C_10_H_20_O_2_ | 172 | 67.576 |
|  | 16 | 23.079 | Vanillin | C_8_H_8_O_3_ | 152 | 0.066 |
|  | 17 | 23.786 | 8-Hydroxycarvotanacetone | C_10_H_16_O_2_ | 168 | 0.064 |
|  | 18 | 25.944 | Methyl laurate | C_13_H_26_O_2_ | 214 | 0.038 |
|  | 19 | 27.009 | Dodecanoic acid | C_12_H_24_O_2_ | 200 |  |
|  | 20 | 27.782 | Diethyl Phthalate | C_12_H_14_O_4_ | 222 | 0.456 |
|  | 21 | 31.127 | Coniferyl aldehyde | C_10_H_10_O_3_ | 178 | 0.051 |
|  | 22 | 31.240 | (E)-Coniferyl alcohol | C_10_H_12_O_3_ | 180 | 0.095 |
|  | 23 | 35.726 | Dibutyl phthalate | C_16_H_22_O_4_ | 278 | 1.056 |
|  | 24 | 43.747 | Docosanal | C_22_H_44_O | 324 | 0.111 |
|  | 25 | 45.189 | Tricosanal | C_23_H_46_O | 338 | 0.055 |
| **Methanol**  **(Embryo)** | 1 | 7.529 | Furfural | C_5_H_4_O_2_ | 96 | 0.883 |
|  | 2 | 7.840 | Furfuryl alcohol | C_5_H_6_O_2_ | 98 | 1.992 |
|  | 3 | 9.536 | 2-Hydroxycyclopent-2-en-1-one | C_5_H_6_O_2_ | 98 | 0.581 |
|  | 4 | 10.007 | Citraconic anhydride | C_5_H_4_O_3_ | 112 | 1.807 |
|  | 5 | 10.639 | 5-Methyl-2-furfural | C_6_H_6_O_2_ | 110 | 0.079 |
|  | 6 | 10.667 | 2,6-Dimethylpyrazine | C_6_H_8_N_2_ | 108 | 0.223 |
|  | 7 | 10.856 | Phenol | C_6_H_6_O | 94 | 0.048 |
|  | 8 | 11.044 | 2,4-Dihydroxy-2,5-dimethyl-3(2H)-furan-3-one | C_6_H_8_O_4_ | 144 | 0.848 |
|  | 9 | 11.421 | 2H-Pyran-2,6(3H)-dione | C_5_H_4_O_3_ | 112 | 0.165 |
|  | 10 | 12.995 | Phenylethanal | C_8_H_8_O | 120 | 0.499 |
|  | 11 | 13.306 | 2-Furoic acid | C_5_H_4_O_3_ | 112 | 0.086 |
|  | 12 | 13.796 | 3,5-Dihydroxy-6-methyl-2,3-dihydro-4H-pyran-4-one | C_6_H_8_O_4_ | 126 | 4.534 |
|  | 13 | 15.474 | 5-Hydroxy-2-methyl-4H-pyran-4-one | C_6_H_6_O_3_ | 126 | 0.051 |
|  | 14 | 17.000 | 5-Hydroxymaltol | C_6_H_6_O_4_ | 142 | 0.417 |
|  | 15 | 17.801 | 4-vinylphenol | C_8_H_8_O | 120 | 1.920 |
|  | 16 | 17.877 | 2-Thiopheneethanol | C_6_H_8_OS | 128 | 1.111 |
|  | 17 | 18.178 | 5-(Hydroxymethyl)-2-furfural | C_6_H_6_O_3_ | 126 | 6.814 |
|  | 18 | 18.640 | 1,2-Diacetylglycerol | C_7_H_12_O_5_ | 176 | 2.194 |
|  | 19 | 20.751 | 4-Vinylguaiacol | C_8_H_8_O | 120 | 3.589 |
|  | 20 | 21.769 | Decanoic acid | C_10_H_20_O_2_ | 172 | 1.413 |
|  | 21 | 26.745 | Dodecanoic acid | C_12_H_24_O_2_ | 200 | 0.774 |
|  | 22 | 26.952 | Vanillic acid | C_8_H_8_O_4_ | 168 | 0.119 |
|  | 23 | 27.160 | 4-Methyl-2,5-dimethoxybenzaldehyde | C_10_H_12_O_4_ | 180 | 4.421 |
|  | 24 | 30.977 | Methyl homovanillate | C_10_H_12_O_4_ | 196 | 0.223 |
|  | 25 | 33.304 | Methyl ferulate | C_11_H_12_O_4_ | 208 | 0.313 |
|  | 26 | 35.397 | Palmitic acid | C_16_H_32_O_2_ | 256 | 0.267 |
|  | 27 | 35.727 | Dibutyl phthalate | C_16_H_22_O_4_ | 278 | 0.080 |
|  | 28 | 38.158 | Methyl sinapate | C_12_H_14_O_5_ | 238 | 0.223 |
|  | 29 | 38.196 | Methyl oleate | C_19_H_36_O_2_ | 296 | 0.149 |
|  | 30 | 38.742 | Linoleic acid | C_18_H_32_O_2_ | 280 | 0.095 |
|  | 31 | 38.827 | Oleic acid | C_18_H_34_O_2_ | 282 | 1.068 |
|  | 32 | 45.189 | Tricosanal | C_23_H_46_O | 338 | 0.045 |
|  | 33 | 53.661 | Bulbocapnine | C_19_H_19_NO_4_ | 325 | 0.232 |

**Table S5 Variance analysis on the effect of environmental factors on germination rate of *Cinnamomum migao* seed.**

| **Treatments** | **Square sum of type III** | **df** | **Mean square** | **F-value** | ***Sig*.** |
| --- | --- | --- | --- | --- | --- |
| Light | 3.429 | 1 | 3.429 | 0.706 | 0.408 |
| Temperature | 1554.286 | 6 | 259.048 | 55.333 | 0.000 |
| Light_*_Temperature | 4.571 | 6 | 0.762 | 0.157 | 0.986 |
